# Macrolide-resistant *Mycoplasma pneumoniae* in South Korea: a strong association with *M. pneumoniae* type 1

**DOI:** 10.1017/S0950268819002000

**Published:** 2019-11-27

**Authors:** Hye-Young Lee, Sang-Ho Choi, Jeonghyun Chang, Mi-Na Kim, Jinho Yu, Heungsup Sung

**Affiliations:** 1 Department of Laboratory Medicine, University of Ulsan College of Medicine and Asan Medical Center, Seoul, Korea; 2 Department of Laboratory Medicine, National Cancer Center, Goyang, Korea; 3 Department of Internal Medicine, University of Ulsan College of Medicine and Asan Medical Center, Seoul, Korea; 4 Department of Laboratory Medicine, University of Inje College of Medicine, Paik Hospital, Goyang, Korea; 5 Department of Pediatrics, University of Ulsan College of Medicine and Asan Medical Center, Seoul, Korea

**Keywords:** Genotyping, macrolide resistance, *Mycoplasma pneumoniae*, outbreak

## Abstract

*Mycoplasma pneumoniae* is a main pathogen causing community-acquired pneumonia in children and young adults. Since the emergence of macrolide-resistant *M. pneumoniae* in the early 2000s in Japan, it has been increasingly reported worldwide as a growing problem in treatment for children. With increasing macrolide-resistant *M. pneumoniae* and limited data regarding its characterization and molecular analysis, we investigated the dominant *M. pneumoniae* strains during the recent outbreak in South Korea, and evaluated if there was an association between a specific type and macrolide resistance. Between October 2014 and December 2016 in South Korea, 249 respiratory specimens obtained from patients with confirmed *M. pneumoniae* pneumonia were genotyped the P1 adhesin gene, and the mutations associated with resistance (A2063G and A2064G) were tested by sequencing the targeted domain V regions of the 23S ribosomal RNA gene. Results revealed that *M. pneumoniae* type 1 were predominant, which was strongly associated with macrolide-resistance during the whole study period. This is the first study assessing whether *M. pneumoniae* subtype is related to macrolide resistance during the outbreak of *M. pneumoniae*.

*Mycoplasma pneumoniae* is a main pathogen causing community-acquired pneumonia (CAP) in children and young adults [1, 2–5]. Epidemic outbreaks occur every 3–7 years, while *M. pneumoniae* infection is endemic worldwide [1, 2]. Macrolides are the first choice of treatment in *M. pneumoniae* infection in children [6, 7]. Since the emergence of macrolide-resistant *M. pneumoniae* in the early 2000s in Japan [2, 8], macrolide-resistant *M. pneumoniae* infection has been increasingly reported in several countries, with prevalence now ranging from 0% to 10% in Europe and the USA, and ~69%–95% in Asia [2–10]. Due to potential risks of fluoroquinolones or doxycycline treatment in children, an increase in macrolide-resistant *M. pneumoniae* infection is a growing problem [3]. With increasing macrolide-resistant *M. pneumoniae* and limited data regarding its characterization and molecular analysis, we investigated the dominant *M. pneumoniae* strains during the recent outbreak in South Korea. Further, we examined whether there were differences between each strain in the presentation of clinical features. We also evaluated if there was an association between a specific type and macrolide resistance.

Between October 2014 and December 2016 in Asan Medical Center, Seoul, Korea, 8375 respiratory samples were obtained from CAP patients, who were diagnosed based on clinical symptoms or radiologic findings. Of these, 622 samples were positive for *M. pneumoniae* using the AmpliSens *Mycoplasma pneumoniae*/*Chlamydophila pneumoniae*-FRT PCR kit (InterLabService Ltd., Moscow, Russia). Of these 622 *M. pneumoniae*-positive samples, 249 samples were available for further testing (Table 1). Typing of *M. pneumoniae* isolates was performed by targeting the P1 adhesin gene with primer pairs as previously documented [6]. To identify major mutations associated with macrolide resistance (A2063G and A2064G), we amplified the domain V regions of the 23S ribosomal RNA gene by methods described previously [5]. Demographic and clinical data for all study populations were collected from electronic medical records. This work was approved by the Institutional Review Board. Informed consent was waived by the Institutional Review Board of Asan Medical Center because this study was performed retrospectively and did not require any extra clinical specimens. Descriptive statistics were performed in terms of quantitative and qualitative data and absolute frequency. Comparisons were conducted using the *χ*^2^ test or Fisher's exact test for categorical variables and Student's *t* test or Mann–Whitney test for continuous variables, as appropriate. A *P* value < 0.05 was defined as statistically significant. SPSS version 18.0 for Windows (SPSS Inc., Chicago, IL, USA) was used for data analysis.

**Table 1.** Patients' demographics and clinical features, according to sequencing type of P1 adhesin gene

Characteristics	Type 1 of P1 gene (*n* = 201)	Type 2 of P1 gene (*n* = 48)	*P* value
Age, years	8.85 ± 11.0	17.2 ± 20.3	<0.001
Sex			
Male, *n* (%)	91 (45.3)	18 (37.5)	0.329
Female, *n* (%)	110 (54.7)	20 (62.5)	
Febrile duration	6.7 ± 3.6	5.3 ± 4.7	0.027
Symptoms duration, *n* (%)			
Fever	197 (98.0)	47 (97.9)	1.000
Cough	197 (98.0)	48 (100)	1.000
Sputum	163 (81.1)	35 (72.9)	0.207
Dyspnea	28 (13.9)	7 (14.6)	0.907
Wheezing	40 (19.9)	9 (18.8)	0.875
Gastrointestinal signs	50 (24.9)	10 (20.8)	0.556
Laboratory findings			
WBC	8592.5 ± 4132.1 (*n* = 186)	8819.1 ± 4063.8 (*n* = 42)	0.748
Haemoglobin	12.3 ± 1.1 (*n* = 186)	12.3 ± 1.5 (*n* = 42)	0.887
Platelet	284.1 ± 106.3 (*n* = 186)	247.1 ± 95.8 (*n* = 42)	0.039
ANC	5497.8 ± 3471.5 (*n* = 186)	5990.1 ± 3673.4 (*n* = 42)	0.431
CRP	7.4 ± 29.7 (*n* = 183)	6.7 ± 6.5 (*n* = 41)	0.882
Radiologic findings, *n* (%)			
Consolidation	122 (60.7)	34 (70.8)	0.133
Interstitial infiltration	55 (27.4)	6 (12.5)	
Consolidation + interstitial pattern	12 (6.0)	4 (8.3)	
Others	11 (5.5)	4 (8.4)	
Previous immunosuppressive drugs, *n* (%)	13 (6.5)	3 (6.3)	1.000
Previous macrolide treatment, *n* (%)	130 (64.7)	11 (35.4)	<0.001
Macrolide resistance mutation, *n* (%)	169 (80.7)	11 (19.3)	<0.001
A2063G	167	11	<0.001
A2064G	2	0	
Disease severity, *n* (%)			0.069
Inpatients	133 (66.2)	25 (52.1)	
Outpatients	68 (33.8)	23 (47.9)	

WBC, while blood cell counts; ANC, absolute neutrophil counts; CRP, C-reactive protein.

*Note*: Data are presented as number (%) or mean ± standard deviation unless otherwise specified.

Overall, 180 (72.3%) of the 249 *M. pneumoniae-*positive specimens harboured mutations in the 23S ribosomal RNA gene. Genotyping revealed that *M. pneumoniae* subtype 1 was more prevalent during the entire outbreak, as follows: October 2014–June 2015, 34 (97.1%) of 35; July 2015–March 2016, 132 (80.0%) of 165; April 2016–December 2016, 35 (71.4%) of 49 (Fig. 1).

**Fig. 1.**
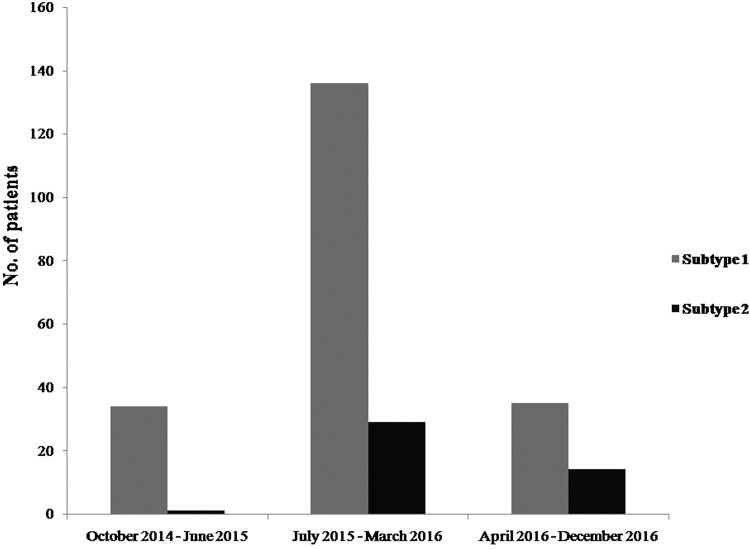
Distribution of P1 adhesion gene subtypes of 249 *M. pneumoniae* between October 2014 and December 2016.

Patients' demographics and clinical features according to sequencing type of P1 adhesin gene were summarised in Table 1. Two hundred and one (80.7%) were classified as type 1 and 48 (19.3%) as type 2. Patients infected with type 1 were younger (8.9 years *vs.* 17.2 years, respectively, *P* < 0.001) and more likely to have longer fever (temperature ≥38 °C) duration (6.7 ± 3.6 days *vs.* 5.3 ± 4.7 days, respectively, *P* < 0.027), compared with patients infected with type 2. The most common clinical symptoms were cough, fever and sputum in both groups (98.4%, 98.0% and 79.5%, respectively). Chest radiographs of all patients were available, and lobar consolidation patterns were most common without statistically significant differences between these two types (60.7% in type 1 *vs.* 70.8% in type 2, *P* < 0.133). Frequencies of each clinical symptoms and laboratory findings, except for platelet counts, were not different between type 1 and type 2 of *M. pneumoniae*. Further, 169 (80.7%) of the type 1 were macrolide-resistant *M. pneumoniae*. Of these, the A2063G mutation was identified in 167 (98.8%), and the A2064G mutation was identified in two (1.2%) patients. Conversely, only 11 (19.3%) of the type 2 were macrolide-resistant *M. pneumoniae*, of which all had the A2063G mutation. The dominant macrolide-resistant genotype was type 1. Given that hospitalised patients have higher disease severity compared to outpatients, we evaluated the proportion of hospitalised patients in each type. The rate of each was 66.2% in type 1 and 52.1% in type 2 (*P* = 0.069).

In this study, a strong association between macrolide resistance and *M. pneumoniae* type 1 was observed. Previous studies attempted to clarify associations between *M. pneumoniae* type and macrolide resistance, but most of them did not determine the association between type and macrolide-resistant *M. pneumoniae* [1, 7]. Only one study in China with 53 clinical isolates documented an association between *M. pneumoniae* strain types and erythromycin resistance [3]. The present study represents the second study with a large group of clinical isolates that demonstrated relatedness between strain type and macrolide resistance. From the clinical data in this study, clinical presentation, laboratory findings and radiologic findings were similar between the groups infected with type 1 and type 2. In addition, our data indicated that type 1 was detected in over 80% of sequenced strains during the epidemic with the co-circulation of type 1 and type 2. Our group reported the substantially increased prevalence of macrolide-resistance of *M. pneumoniae* in children ranging from 2.9% in 2003 to 62.9% in 2011 [10]. Together with our data of a 76.3% macrolide-resistant *M. pneumoniae* rate during 2014–2016, the prevalence of macrolide-resistant *M. pneumoniae* increased continuously over 10 years. Even though most strains isolated in this study were type 1 of the P1 adhesin gene, it is still unclear whether the macrolide-resistant *M. pneumoniae* isolates originated from the same clone. Therefore, further studies regarding the isolate spread are necessary.

In summary, the dominant clinical strain of *M. pneumoniae* during a recent outbreak was type 1, which was more likely to infect younger patients. Given the current rapidly increasing trend of macrolide-resistant *M. pneumoniae* incidence, the continuing epidemiological monitoring of macrolide resistance is necessary to recognise macrolide resistance strains early and complement effective care against these infections.
